# Complete Genome Sequences of Three African Foot-and-Mouth Disease Viruses from Clinical Samples Isolated in 2009 and 2010

**DOI:** 10.1128/genomeA.00326-16

**Published:** 2016-05-05

**Authors:** Steven Van Borm, Toon Rosseel, Andy Haegeman, Mpolokang Elliot Fana, Latoa Seoke, Joseph Hyera, George Matlho, Frank Vandenbussche, Kris De Clercq

**Affiliations:** a Molecular Platform, Veterinary and Agrochemical Research Centre, Ukkel, Belgium; b Laboratory for Vesicular and Exotic Diseases, Veterinary and Agrochemical Research Centre, Ukkel, Belgium; c Botswana Vaccine Institute, Gaborone, Botswana

## Abstract

The complete genome sequences of three foot-and-mouth disease viruses (one virus of each serotype SAT1, SAT2 and O) were directly sequenced from RNA extracted from clinical bovine samples, demonstrating the feasibility of full-genome sequencing from strong positive samples taken from symptomatic animals.

## GENOME ANNOUNCEMENT

In Africa, foot-and-mouth disease (FMD) is a primary livestock disease of economic importance, affecting wild and domestic cloven-hoofed animals (1). The etiological agent, FMD virus (FMDV) (*Picornaviridae*, *Aphthovirus*), exists in seven distinct serotypes (Euro-Asiatic serotypes O, A, C, and Asia 1, and South African territories [SAT] 1 to 3), with multiple subtypes (2). 

To evaluate the feasibility of complete genome sequencing from clinical samples using unbiased RNA sequencing (RNA-seq) methods, three FMDV strongly positive epithelial samples from symptomatic cattle were selected using real-time reverse transcription-PCR (RT-PCR) (3). From these samples, originating from Zambia and Namibia, three FMDV isolates (SAT2/ZAM18/2009, O/ZAM14/2010, and SAT1/NAM01/2010) were obtained and characterized (using virus isolation, antigen enzyme-linked immunosorbent assay [ELISA], and partial sequencing). The original clinical samples were homogenized in phosphate-buffered saline (10% [wt/vol]), pretreated by 0.45 µM size selective filtration and nuclease, and RNA was extracted as previously described (4, 5). cDNA was synthesized using SuperScript III reverse transcriptase (Thermo Fisher Scientific) and random hexamer primers, according to the manufacturer’s instructions. Sequencing libraries were prepared using 1 ng (or the maximum amount available) of cDNA and the Nextera XT kit (Illumina), according to the manufacturer’s instructions, quantified with the library quantification kit Illumina platforms (Kapa Biosystems), and fragment length distribution was verified using the Bioanalyzer with the high-sensitivity DNA kit (Agilent Technologies). Sequencing was performed using a MiSeq reagent kit version 3 (Illumina) with 2 × 300-bp paired-end sequencing. Twenty-three libraries were multiplexed using standard Illumina indexing primers.

The quality of the sequences was checked with FastQC version 0.10.1 (http://www.bioinformatics.babraham.ac.uk/projects/fastqc/). Stretches containing unidentified nucleotides (“N”) were trimmed using Cutadapt version 1.3 (6) prior to quality trimming using Sickle version 1.210 (*Q* score, <30; length, <50 bp) (7). *De novo* assembly was performed using Newbler version 2.9 (Roche). The protein-coding genes were predicted relative to sequences with accession numbers AF540910, HM191257, and AY593842 using GATU (8).

The complete genome sequences of SAT2/ZAM18/2009 and O/ZAM14/2010 were obtained, with average coverages of 2,151× and 732×, respectively. These FMDV genomes contain a single open reading frame (ORF) of 7,008 (SAT2/ZAM18/2009) and 6,999 nucleotides (nt) (O/ZAM14/2010) encoding a polypeptide precursor protein, and they share high nucleotide homology with AF540910 and HM191257, respectively. The contig representing SAT1/NAM01/2010 contains a single 7,020-nt ORF (polypeptide precursor protein) and shares a high nucleotide homology with AY593842. As only a limited number of FMDV reads were available for the latter sample, two gaps (a 67-nt gap around the poly(C) tract and a 110-nt gap centered around contig position 6000) were closed using PCR amplification and Sanger sequencing, while the average coverage was <10×.

These data demonstrate the feasibility of direct sequencing of complete FMDV coding sequences from samples from symptomatic animals (real-time RT-PCR threshold cycle [*C_T_*] range of 14.63 to 16.18 for the samples used in this study) using an unbiased cDNA sequencing approach. However, targeted approaches using FMDV-specific cDNA synthesis primers (9) or PCR amplification may result in a better sensitivity for whole-genome sequencing.

### Nucleotide sequence accession numbers.

The complete coding sequences for SAT2/ZAM18/2009, O/ZAM14/2010, and SAT1/NAM01/2010 were assigned DDBJ/EMBL/GenBank accession numbers KU821590 to KU821592.
